# Assessment of post extraction complications in Indians

**DOI:** 10.6026/973206300171120

**Published:** 2021-12-31

**Authors:** Keerthika Saravanan, MP Santhosh Kumar

**Affiliations:** 1 Saveetha Dental College and Hospitals, Saveetha Institute of Medical and Technical sciences, Saveetha University, Chennai, India

**Keywords:** Extraction, Caries, Systemic disease, Dry socket, smoking, complications, trismus, pain

## Abstract

Extraction is one of the commonest procedures in dentistry. Therefore, it is of interest to evaluate the post extraction complications in patients undergoing extractions of permanent teeth. A total of 70 adult patients who had undergone dental extractions and presented with post -operative complications were included in the study and evaluated. Data collected was statistically analyzed using SPSS software and results obtained. Most of the patients with post extraction complications were in the age group of 31-40 years (21.6%), followed by 21-30 (20.2%) and 61-70 years (20.2%). Dry socket (39.19%) was the common post extraction complication in our study especially in the age group of 31-40 years. There was a statistically significant association between age of the patients and the post extraction complications (p<0.001). In our study, post extraction complications were commonly observed in age group of 31-40 years with a predilection for males. Dry socket was the most common post extraction complication. Age of the patient has a significant effect on post extraction complications. However, gender, smoking habits and systemic diseases have no influence on post extraction complications.

## Background:

Commonest and oldest oral surgical procedure is extraction [1]. Extraction of tooth requires separation of its attachments to alveolar bone via crystal and principal fiber of periodontal ligament [2]. Since tooth extraction is a routine dental procedure, complications during or after tooth extraction are anticipated [3]. Common reasons for tooth extraction are advanced pulpal and periodontal problems, periapical pathology, and third molar impaction [4]. Difficulties and accidents occurring during extraction interfere with post extraction healing [5,6]. Most frequent complications of tooth extraction include dry socket (4.6%) [7,8]. Rare complications include Oroantral fistula (0.008-0.25%), maxillary tuberosity fracture (0.6%) and mandibular fracture (0.0049%) [9]. Many clinical conditions that influence post extraction healing include diabetes mellitus, HIV, anemia, malnourishment, radio-chemotherapy, immune suppression, prolonged corticosteroid therapy, disorders involving liver, kidney and thyroid organs [10]. Other factors influencing are age, gender, smoking, type of impaction, surgical technique, surgeons experience, peri operative antibiotics, topical antiseptics, intra socket medications and anesthetic techniques [11,12]. Systemic disease on healing of post extraction wounds is known [7,9- 12]. Therefore, it is of interest to document the assessment of post extraction complications.

## Materials & Methods:

This retrospective study was conducted in a private dental college, Chennai, to evaluate the post extraction complications of permanent teeth among dental patients treated from June 2019 to March 2020. The study was initiated after approval from the institutional review board. (SDC/SIHEC/2020/DIASDATA/0619-0320) Pros of the study included enormous digital data, and less time consumption for retrieval of data. Cons of the study included that study population is limited to a certain geographic location. A total of 70 adult patients who had undergone dental extractions and presented with post -operative complications were included in the study. Demographic details like age, gender, systemic diseases, presence of smoking habits and post extraction complications were recorded. Post-extraction complications like pain, swelling, trismus, dry socket, ulcers, paresthesia and bleeding were also recorded, statistically analyzed using Statistical Package for Social Sciences for Windows, version 23.0; Chi-square test was used and results obtained.

## Results:

**Figure 1:**
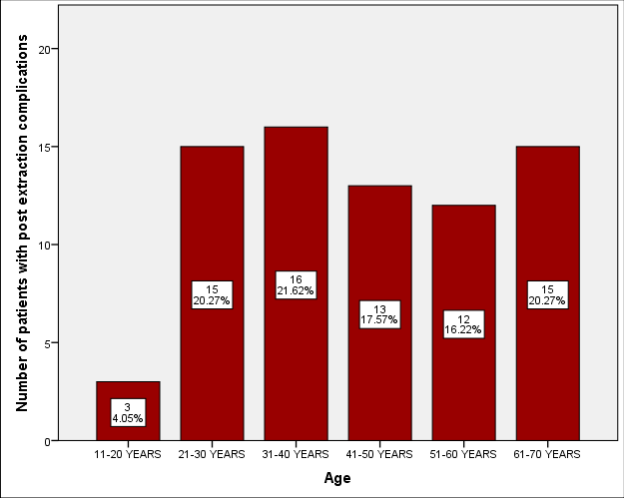
Bar graph depicting age distribution of patients with post extraction complications. X-axis indicates age of the patients in years; Y-axis indicates number of patients with post extraction complications. Most of the patients in the age group 31-40 years (21.62%) had post extraction complications.

**Figure 2:**
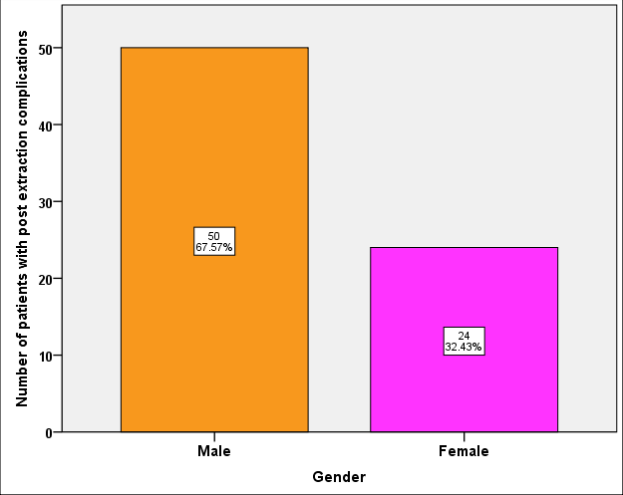
Bar graph depicting gender wise distribution of patients with post extraction complication. X-axis indicates Gender of the patients; Y-axis indicates number of patients with post extraction complications. Post Extraction complications predominantly occurred in males (67.5%) than in females (32.4%).

**Figure 3:**
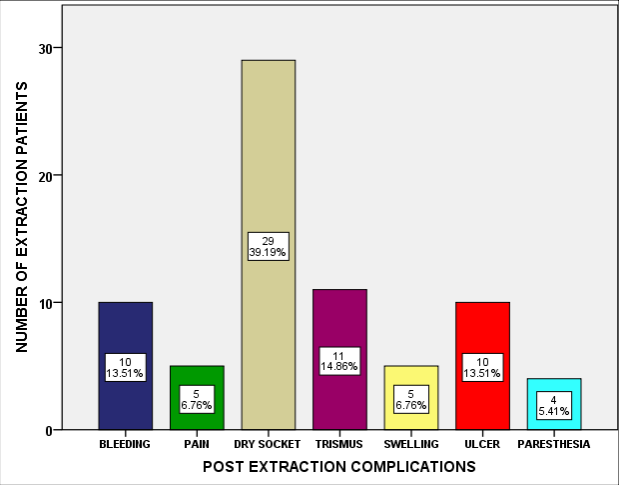
Bar graph depicting distribution of post extraction complications among patients. X-axis - denotes post extraction complications; Y-axis - number of extraction patients. Dry socket (39.19%) was the common post extraction complication, which occurred in our study population.

**Figure 4:**
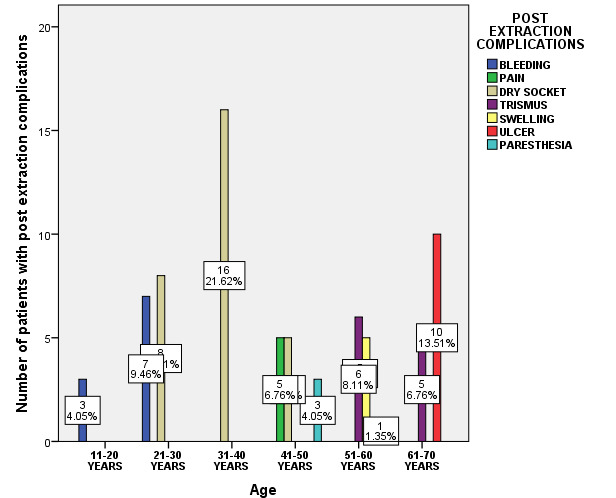
Bar chart showing association between age of the patients and type of post extraction complication. X-axis - age group in years; Y-axis number of patients with post extraction complications. (Pearson Chi-square value = 183.3; p=0.001 < 0.05). Age has an influence on the post extraction complications. Dry socket was more prevalent among the patients of age group 31-40 years.

**Figure 5:**
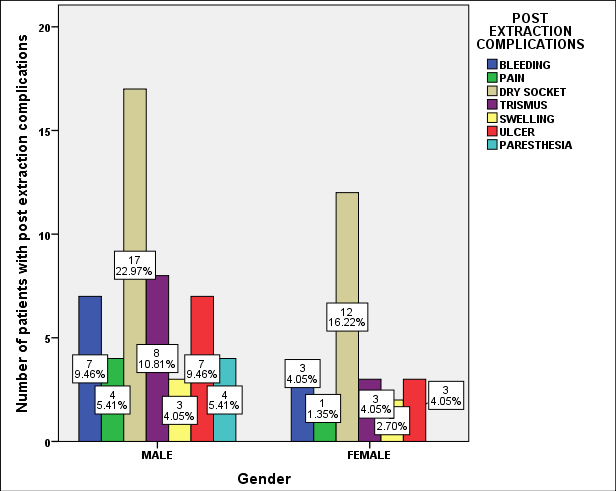
Bar chart showing association between gender of the patients and post extraction complications. X-axis indicates Gender; Y-axis indicates percentage of patients with post extraction complications. (Pearson Chi-square value = 3.650; p=0.724 > 0.05). Gender has no influence on the post extraction complications. In male patients, post extraction complications were Bleeding (9.46%), pain (5.41%), Dry socket (22.9%), Trismus (10.8%), swelling (4.05%), ulcer (9.46%) and paresthesia (5.41%). Among female patients, postoperative complications were Bleeding (4.05%), pain (1.35%), Dry socket (16.22%), Trismus (4.05%), swelling (2.7%), and ulcer (4.05%) and the results were statistically not significant.

**Figure 6:**
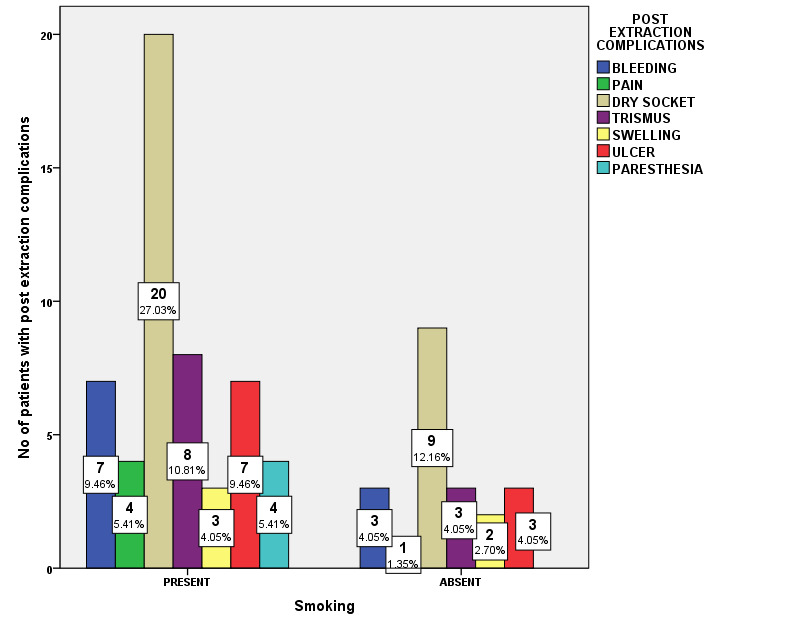
Bar chart showing association between smoking and post extraction complications. X-axis indicates smoking habit; Yaxis indicates number of patients with post extraction complications. (Pearson Chi-square value = 4.389; p=0.624 > 0.05). Smoking has no influence on the post extraction complications. Among patients with smoking habits, Dry socket (27.03%), and other post extraction complications occurred predominantly, than in non-smoking patients - dry socket (12.16%). However, the results were statistically not significant.

**Figure 7:**
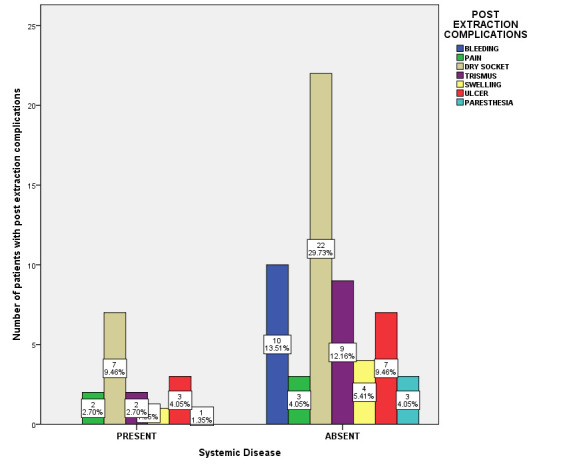
Bar chart showing association between Systemic diseases of patients and post extraction complications. X-axis indicates Systemic diseases; Y-axis indicates number of patients with post extraction complications. (Pearson Chi-square value = 4.389; p=0.624 > 0.05). Systemic disease has no influence on the post extraction complications. Among patients without systemic diseases, Dry socket (29.7%) and other post extraction complications were observed predominantly than in patients with systemic diseases - dry socket (9.46%). However, the results were statistically not significant.

In our study, the mean age group of participants in the study was 42 yrs. Most of the patients with post extraction complications were in the age group of 31-40 years (21.6%), followed by 21-30 (20.2%) and 61-70 years (20.2%). Least prevalence of post extraction complications was observed in 11-20 years (4%). In our study peak incidence of post extraction complications was observed in the age group between 31-40 years (Figure 1). In our study, 67.5% were male patients and 32.4% were female patients. Post Extraction complications predominantly occurred in males than in females (Figure 2). Among various post extraction complications, bleeding (13.5%), pain (6.76%), dry socket (39.19%), trismus (14.86%), swelling (6.765), ulcer (13.51%) and paresthesia (5.41%) was observed in our study. Dry socket (39.19%) was the common post extraction complication, which occurred in our study population (Figure 3). Association between age of the patients and post extraction complications was evaluated. Among various post extraction complications, bleeding (4.05%) in the 11-20 years, Dry socket (21.62%) in 31-40 years and 10.81% in 21-30 years, pain (6.76%) in 41-50 years, trismus (8.11%) in 51-60 years and ulcer (13.51%) in 61-70 years were observed predominantly (Pearson Chi-square value = 183.3; p=0.000). Thus, there was a statistically significant association between age of the patients and the post extraction complications; and age has an influence on the post extraction complications. Dry socket was more prevalent among the patients of age group 31-40 years (Figure 4). Association between gender of the patients and post extraction complications was evaluated. Among male patients post extraction complications were Bleeding (9.46%), pain (5.41%), Dry socket (22.9%), Trismus (10.8%), swelling (4.05%), ulcer (9.46%) and paresthesia (5.41%). Among female patient postoperative complications were Bleeding (4.05%), pain (1.35%), Dry socket (16.22%), Trismus (4.05%), swelling (2.7%), and ulcer (4.05%) (Pearson Chi-square value =3.650; p=0.724). Thus, there was no statistically significant association between gender of the patients and the post extraction complications; and gender has no influence on the various post extraction complications. Male predominance was observed in various post extraction complications; Bleeding (9.46%), pain (5.41%), Dry socket (22.9%), Trismus (10.8%), swelling (4.05%), ulcer (9.46%) and paresthesia (5.41%), Which was statistically not significant (Figure 5). Association between smoking habit and post extraction complications was evaluated. Among smokers Bleeding (9.46%), pain (5.41%), Dry socket (27.03%), Trismus (10.8%), swelling (4.05%), ulcer (9.4%) and paresthesia (5.41%) was observed. Among non-smokers Bleeding (4.05%), pain (1.35%), Dry socket (12.16%), Trismus (4.05%), swelling (2.7%) and ulcer (4.05%) was observed. (Pearson Chi-square value = 4.389; p=0.624). Thus, there was no statistically significant association between smoking habits of the patients and the post extraction complications; and smoking has no influence on the post extraction complications. Among patients with smoking habits, Dry socket (27.03%), and other post extraction complications occurred predominantly, than in non-smoking patients - dry socket (12.16%). However, the results were statistically not significant (Figure 6). Association between systemic diseases of the patients and post extraction complications was evaluated. Among patients with systemic disease Bleeding (13.5%), pain (4.05%), Dry socket (29.7%), Trismus (12.1%), swelling (5.41%), ulcer (9.4%) and paresthesia (4.05%) was observed. Among patients without systemic disease Bleeding (13.5%), pain (4.05%), Dry socket (29.7%), Trismus (12.1%), swelling (5.41%), ulcer (9.4%) and paresthesia (4.05%) was observed (Pearson Chi-square value = 4.389; p=0.624). Thus, there was no statistically significant association between systemic diseases of the patients and the post extraction complications; and systemic disease has no influence on the post extraction complications (Figure 7).

## Discussion:

In our study, peak incidence of post extraction complications was observed in the age group between 31-40 years. This is contradictory to the study by Jaafar et al in which predominant incidence of post extraction complications was reported in the age group between 18-33 years [13]. There is a distinct association between age and postoperative complications. It was reported that in elderly people there was an increased incidence of complications [14]. According to our study, post extraction complications were predominantly present in males (67.57%) than in females (32.43%). These findings were contradictory to study by Monaco et al. which shows higher incidence in the female group. They reported that incidence of postoperative edema (swelling) in female patients (12.7%) was significantly higher when compared to male patients (1.4%) [15]. In the current study the various post extraction complications observed were bleeding (13.5%), pain (6.76%), dry socket (39.19%), trismus (14.86%), swelling (6.765), ulcer (13.51%) and paresthesia (5.41%). Dry socket (39.19%) was the common post extraction complication in our study population. This is in accordance to the study by Sigrun GR et al in which the common complications after third molar removal were alveolar osteitis (4.2%) followed by sensation disorder (1.5%) [16]. Adeyemo WL et al in their study reported 8.2% cases of localized osteitis, 1.6% cases of acutely infected alveolus and 1.2% of patients with acutely inflamed alveolus mostly after molar extractions with a greater female predilection [17]. In our study no significant association was found between risk factors like smoking, systemic disease with post extraction complications. These reports were contradictory to several studies, which demonstrated correlation between smokings with post extraction complications. It was reported that cigarette smoking reduces postoperative socket filling with blood, impairing wound healing [16-18]. Drawback of our study is smaller sample size from a specific geographical location. Future scope of the study is that further longitudinal prospective multicenter studies are required to prove the hypotheses.

## Conclusion:

Data shows that post extraction complications were mostly observed in age group of 31-40 years with a predilection for males. Dry socket was the most common post extraction complication. Age of the patient has a significant effect on post extraction complications. However, gender, smoking habits and systemic diseases have no influence on post extraction complications.

## Author contributions:

The study was conducted and written by S Keerthika. Santhosh Kumar supervised the study and corrected the manuscript.

## References

[1] Abhinav RP (2019). Ann Maxillofac Surg..

[2] Patturaja K, Pradeep D (2016). Research J. Pharm. and Tech..

[3] Rao TD, Santhosh Kumar MP (2018). Research J. Pharm. and Tech..

[4] Packiri S (2017). J Clin Diagn Res..

[5] Jesudasan JS (2015). Br J Oral Maxillofac Surg..

[6] Santhosh Kumar MP (2017). Asian J Pharm Clin Res..

[7] Santhosh Kumar MP (2017). Asian J Pharm Clin Res..

[8] Rahman R, Santhosh Kumar MP (2017). Asian J Pharm Clin Res..

[9] Santhosh Kumar MP, Rahman R (2017). Asian J Pharm Clin Res..

[10] Vijayakumar Jain S (2019). J Maxillofac Oral Surg..

[11] Christabel A (2016). Int J Oral Maxillofac Surg..

[12] Santhosh Kumar MP (2016). Asian J Pharm Clin Res..

[13] Jaafar N (2000). Singapore Dent J..

[14] Oginni FO (2008). J Oral Maxillofac Surg..

[15] Monaco G (2015). J Oral Maxillofac Surg..

[16] Sigron GR (2014). Swiss Dent J..

[17] Adeyemo WL (2007). J Contemp Dent Pract..

[18] Sanari AA (2020). Saudi Dent J..

